# Analysis of T‐cell receptor repertoire in peripheral blood of patients with pancreatic cancer and other pancreatic diseases

**DOI:** 10.1111/jcmm.16358

**Published:** 2021-03-08

**Authors:** Hui Wang, Yue Yuan, Chenglin Lu, Siqi Zhou, Yixuan Zhang, Jing Zhao, Chenghu Xu, Jie Yang, Haochen Su, Borui Li, Xihan Li, Pin Wang, Guifang Xu, Lei Wang, Xiaoping Zou, Shanhua Bao, Shu Zhang, Ying Lv

**Affiliations:** ^1^ Department of Gastroenterology Nanjing University Medical School Affiliated Drum Tower Hospital Nanjing China; ^2^ Department of Gastroenterology Nanjing Medical University Affiliated Drum Tower Clinical Medical College Nanjing China; ^3^ Department of General Surgery The Afflicted Drum Tower Hospital Nanjing University Medical School Nanjing China; ^4^ Department of Gastroenterology Jiangsu University Affiliated Drum Tower Hospital Nanjing China

**Keywords:** high‐throughput sequencing, immunotherapy, pancreatic cancer, pancreatic disease, T‐cell receptor repertoire

## Abstract

Pancreatic cancer (PC) has been the fourth cancer‐related death worldwide, diagnosed at an unresectable stage due to its rapid progression and few symptoms of this disease at early stages. The aim of this study was to determine the association between the diversity of T‐cell receptor (TCR) repertoire and clinicopathological characteristics of patients with PC and other benign pancreatic diseases. In order to make a comprehensive analysis the TCR repertoire, high‐throughput sequencing was used to differentiate complementarity determining region 3 (CDR3) of the TCR β chain in peripheral blood samples from 3 PC, 3 chronic pancreatitis, 3 pancreatic cystic lesions and 3 pancreatic neuroendocrine tumour patients. We found that there were significant differences related to TCR repertoire between PC and other pancreatic diseases, and PC is a relatively immunosuppressive tumour. Changes of peripheral TCR repertoire may be used to predict the progression of PC and the response to immunotherapy. And there may exist novel‐specific antigens in PC patients which could be used to design targeting immunotherapy in the nearly future.

## 1 INTRODUCTION

Pancreatic cancer (PC) has been the fourth cancer‐related death worldwide,^1^ with only a 9% chance of survival for more than five years. PC patients are usually diagnosed at an unresectable stage due to its rapid progression and few symptoms of this disease at early stages. Thus, the early detection of this fatal disease and the development of new treatment strategies are extremely necessary.^2^

The dysfunction of immune cells, especially T cells, is involved in cancer progression of various solid tumours. Immunotherapeutic methods, such as the immune checkpoint inhibitors targeting cytotoxic T lymphocyte‐associated protein 4 (CTLA‐4) or programmed cell death 1 (PD‐1) / programmed cell death 1 ligand (PD‐L1), have made a breakthrough in improving the survival rate of cancer patients, which makes people pay more and more attention on the anti‐tumour properties of the immune system.^3^, ^4^, ^5^ The killing effect of T cells on cancer cells through immunotherapy is similar to that of traditional treatments (such as chemotherapy and radiotherapy), making T cells crucial factors in anti‐cancer immune response.^6^, ^7^ Therefore, in order to accurately understand the individual tumour immunity, high‐throughput analysis of T cells is necessary.^8^

Evidence have shown that T cells are indispensable cells in the development of PC, chronic pancreatitis (CP),^9^, ^10^, ^11^, ^12^ pancreatic neuroendocrine tumours (PNET)^13^, ^14^, ^15^, ^16^, ^17^ and other pancreatic diseases. They recognize the antigenic peptides presented by the major histocompatibility complex (MHC) through the specific T‐cell receptors (TCR) on the cell membrane. The TCR repertoire is mainly composed of α and β chains, and its diversity is mainly determined by the random combination of variable (V) and linked (J) gene fragments of these two chains, reflecting the adaptive immune status. Complementary determination region 3 (CDR3) determines the specificity and diversity of TCR through V (D) J recombination coding. Therefore, high‐throughput sequencing of the TCR CDR3 region may be helpful for us to understand the process of carcinogenesis and anti‐cancer immune mechanism.

Recently, a series of researches have demonstrated that TCR CDR3 diversity might be used as a good biomarker, which may be helpful for early diagnosis, monitoring of curative effect and evaluation of prognosis.^8^, ^9^, ^10^, ^11^, ^12^, ^13^, ^14^, ^15^, ^16^, ^17^, ^18^, ^19^, ^20^, ^21^, ^22^ Our understanding of the TCR characteristics in PC is very limited. We find that, like other malignant tumours, the TCR repertoire of PC is heterogeneous in different regions within the same tumour.^23^ Besides, for patients with advanced malignancies, tumour tissues are difficult to obtain. Thus, using blood samples from these patients is a better option to overcome these problems. As we all know, PC can sometimes hardly be distinguished with other pancreatic benign diseases, such as pancreatic cystic lesions (PCL), PNET and CP. Thus, we must clearly clarify the differences of TCR diversity among diseases at high risk or suspected of PC, so that TCR diversity in peripheral blood samples can be used to guide disease surveillance and treatment.

Therefore, in this study, we made a systematic analysis for the first time to compare the diversity of the β chain of TCR CDR3 in the peripheral blood samples of patients with PC and other benign pancreatic diseases, in order to determine the association between the diversity of TCR repertoire and clinicopathological features of these patients.

## 2 MATERIALS AND METHODS

### 2.1 Patients and specimens

Four groups of PC (3), CP (3), PCL (3) and PNET (3) patients with complete clinical information and available peripheral blood samples in Drum Tower Hospital affiliated to Nanjing University Medical School were enrolled in this study. 3 PC patients in our study had undergone surgeries in the General Surgery Department in our hospital. This study was approved by the Ethics Committee of Drum Tower Hospital affiliated to Nanjing University Medical School. All clinicopathological characteristics, including age, gender, tumour stage, alcohol intake, smoking history and prognostic information were retrospectively collected from the patient records. All the data collection, data analysis and experiments were carried out in accordance with the approved protocol.

### 2.2 RNA extraction and TCR library preparation

The total RNA extraction kit (TIANGEN) was used to extract total RNA from lymphocytes in strict accordance with the operation steps in the instructions. We obtained mononuclear cells from 2ml peripheral blood using the TRIzol reagent according to the manufacturer's protocol. The concentrations of RNA were evaluated using a NanoDrop ND‐2000 spectrophotometer (Thermo Scientific). A total of 200ng RNA was converted into cDNA by reverse transcription using a Transciptor First Strand cDNA Synthesis Kit (Roche Applied Science, Penzberg, Germany) according to the manufacturer's protocol on a T100TM Thermal Cycler (Bio‐Rad Inc).^24^ Quality inspection was carried out with Agilent2200, and cDNA was stored at −80°C.

Then, the libraries were constructed following the protocol from Liang et al.^25^ For TCRβ CDR3 library preparation, two‐round nested amplicon arm‐PCR was performed with a Multiplex PCR Assay Kit Ver.2 (TaKaRa) using specific primers against each variable and constant gene. PCR products were purified by agarose gel electrophoresis, amplified using Illumina sequencing primers with different sample barcodes and subjected to high‐throughput sequencing using the Illumina HiSeq X Ten PE150 platform.

### 2.3 High‐throughput sequencing of TCR and CDR3 sequencing analysis

High‐throughput sequencing in TCR‐beta CDR3 region using Illumina HiSeq X Ten platform is a previously verified method. The reference sequence of V and J genes was downloaded from IMGT database (http://www.imgt.org/vquest/refseqh.html). Using blastn (version2.7.1) in blast + software to compare the sequencing data to the reference sequence. The frequency of V, J gene in all samples was counted, and bar charts and pie charts were drawn according to the frequency of V and J genes top 20. Besides, the heatmap was drawn by using heatmap package in R language. According to the use of V‐J combination of each sample, the V‐J recombination circle diagram was drawn. The location information of the gene is taken from the human genome annotation file on the NCBI website. Then, we standardized the data by converting the V‐J pair frequency of each sample into rpm (reads per million), and then used the data for t‐test to get p‐value and calculate the Fold Change to draw the volcano map. The D gene segment is located between V region and J region. For the reads, aligned to V region and J region, it is truncated from the end of V region to the beginning of J region, and this part of the sequence is compared with the D region sequence in IMGT database. Also use blastn (version2.7.1) in blast + software. The CDR3 region was identified using blastn in BLAST+ (version2.7.1) by aligning to the above reference sequence of V and J genes. The diversity of TCRβ was measured by normalized Shannon diversity entropy which has been widely used for assessing the richness and diversity of TCR as previously described.^26^, ^27^ Circular plots were created using Circos (version 0.69‐6)^28^ to reflect the recombination of jumping pairs.

### 2.4 Statistical analysis

One‐way ANOV A was performed using R to identify the different genes expressed between each group. A 2‐sided *P*‐value < .05 was considered statistically significant. Other results did not include hypothesis testing.

## 3 RESULTS

### 3.1 Patient characteristics

A total of 12 patients were enrolled in this study (7 females and 5 males), including 3 cases of PC (group A), 3 cases of CP (group B), 3 cases of PCL (group C) and 3 cases of PNET (group D). The age of these patients ranged from 30 to 70 years old. Younger than other groups, all of the CP groups were under 40 years old. According to the medical records, 2 patients had cholecystitis history, 3 patients had uterine leiomyoma, 1 patient had high blood pressure, 1 patient had type 2 diabetes, 1 patient had hyperthyroidism and 1 patient had chronic hepatitis B infection (Table 1). Nine patients went to the hospital because of the corresponding symptoms. Most (6/9) of the lump were located in the head of the pancreas. Detailed clinicopathological characteristics were presented in Table 1. All cancer patients were finally diagnosed after surgical resections, but none of them had received chemotherapy or radiotherapy before surgeries.

**TABLE 1:** Clinicopathological characteristics for the included patients

Patient	Gender	Age (year)	Size of tumor (mm)	Size of tumor	Pathology	Stage of tumor	Sample	Clinical symptoms	Chronic medical history
A‐1	Female	65	40 × 40 × 30	Head of pancreas	Adenocarcinoma IB	T2N0MO	Blood	Abdominal pain	Cholecystitis
A‐2	Male	58	40 × 40 × 30	Neck and body of pancreas	Adenocarcinoma IB	T2N0MO	Blood	Asymptomatic	Diabetes
A‐3	Male	68	50 × 50 × 30	Head of pancreas	Adenocarcinoma IIA	T3N0MO	Blood	Asymptomatic	Hypertension
B‐1	Male	33	NA	‐	Chronic pancreatitis	‐	Blood	Abdominal distension	NA
B‐2	Male	37	NA	‐	Chronic pancreatitis	‐	Blood	Abdominal distension	NA
B‐3	Male	37	NA	‐	Chronic pancreatitis	‐	Blood	Abdominal pain	Chronic hepatitis B
C‐1	Female	63	The thickness of the wall:3:8	Head of pancreas	PNET G2	‐	Blood	Asymptomatic	NA
C‐2	Female	42	30 × 30 × 30	Tail of pancreas	PNET G2	‐	Blood	Low blood sugar	Hyperthyroid ism
C‐3	Female	52	20 × 20	Head of pancreas	PNET G1	‐	Blood	Dizziness and fatigue	Uterine leiomyoma
D‐1	Female	50	13.9 × l6.2; 32 × 28	Head of pancreas; Tail of pancreas	SCN	–	Blood	Asymptomatic	Uterine leiomyoma
D‐2	Female	51	25.4 × 25.6	Body and tail of pancreas	SCN	‐	Blood	Abdominal distension	Uterine leiomyoma
D‐3	Female	70	I l.3 × 6.6; 3.9 × 3.6	Head of pancreas; Body of pancresas	SCN	–	Blood	Abdominal pain	Cholecystitis

Abbreviations: PNET, pancreatic neuroendocrine tumors; SCN, pancreatic serous cystadenoma.

### 3.2 The distribution of the high‐frequency fragments of TCRβ CDR3 genes transcripts

**FIGURE 1:**
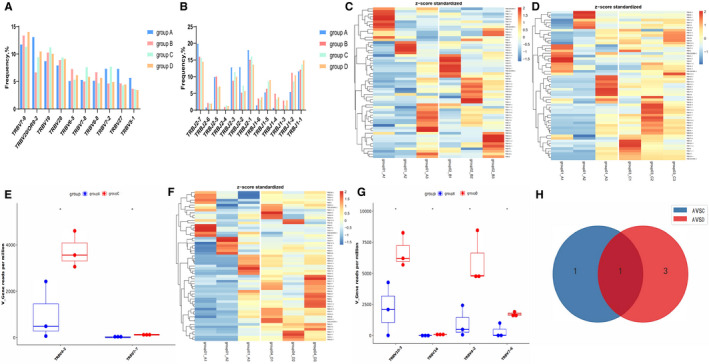
(A, B) Histogram comparison of high‐frequency Vβ, Jβ gene segments among four groups. (C, D, F) Heatmap of statistically significant differential Vβ gene segments expressed in group A (PC) VSB (CP), group A (PC) VSC (PCL) and group A (PC) VS D (PNET). The use frequency of Vβ gene fragments was shown by heat map bar (log2(FC) > 1, *P* <.05). (E, G) Box diagram of differentially expressed Vβ gene segments in group A VS C and group A VS D (*P* <.05). (I) Wayne diagram of meaningful differential Vβ genes between groups. The coincident gene is TRBV4‐2

The nucleotide and amino acid sequences of immune T cells in IMGT database were used as references in 12 samples, and the off‐machine reads were compared according to the way described in the method section. The types and frequencies of all V and J gene fragments in 4 groups were analysed. As Figure S1 depicted, the top ten TCR Vβ and Jβ genes were listed, respectively, according to their frequency. It was noteworthy that most of the high‐frequency fragments in PC, CP, PCL and PNET were coincident (Figure S1 and Figure 1A,B). The sequence of CDR3 was determined by the base position of Vβ and Jβ gene fragments and VDJ recombination. In order to further reveal the characteristics of TCRβ CDR3 between PC and other pancreatic diseases, we next compared the diversity of Vβ, Jβ and Vβ‐Jβ paired genes between them.

### 3.3 Analysis and comparison of differential genes of Vβ, Jβ gene segments and V‐J, V‐D‐J gene combinations among four groups

**FIGURE 2:**
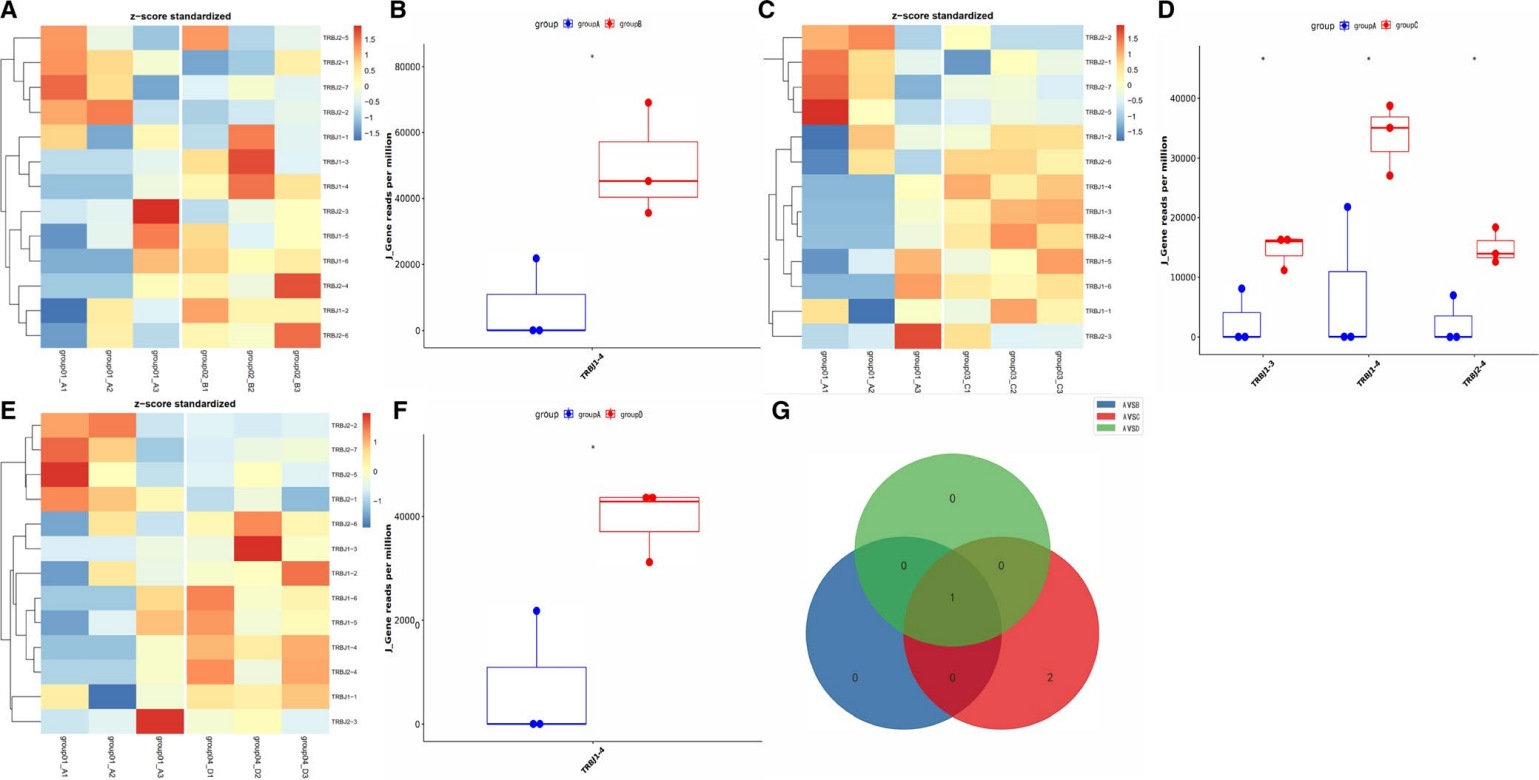
(A, C, E)Heatmap of statistically significant differential Jβ gene segments expressed in group A VS B, group A VS C and group A VS D. The use frequency of Jβ gene fragments were shown by heatmap bar (log2(FC) > 1, *P* <.05). (B, D, F) Box diagram of differentially expressed Jβ gene segments in group A VS B, group A VS C and group A VS D (*P* <.05). (G) Wayne diagram of meaningful differential Jβ genes between groups. The coincident gene is TRBJ1‐4

The comparison of all Vβ and Jβ gene fragments among the four groups was shown in the Figure 1 and Figure 2. There was no significant difference in the frequency of Vβ gene between PC and CP (Figure 1C), while the frequency of some fragments of Vβ gene (TRBV4‐2, TRBV7‐7) in PC was lower than that of PNETs (Figure 1D,E), and the frequency of some fragments of Vβ gene (TRBV16, TRBV7‐6, TRBV4‐2 and TRBV10‐3) in PC was lower than that of PCLs (Figure 1F,G). These data indicated that the expression of the Vβ gene TRBV4‐2 was down‐regulated in PC patients compared with other pancreatic diseases (Figure 1I).

Regarding Jβ genes expression, the frequency of TRBJ1‐4 gene fragment in PC was lower than that in CP (Figure 2A,B), as well as in PCL group (Figure 2E,F). Besides, the frequency of some gene fragments (TRBJ13, TRBJ2‐4 and TRBJ1‐4) in PC was lower than that in PNET (Figure 2C,D). Collectively, the expression of the Jβ gene TRBJ1‐4 was down‐regulated in PC patients compared with other pancreatic diseases (Figure 2G). In general, there were significant differences in the frequencies of some gene fragments of Vβ or Jβ gene between PC and other pancreatic diseases (*P* <.01).

**FIGURE 3:**
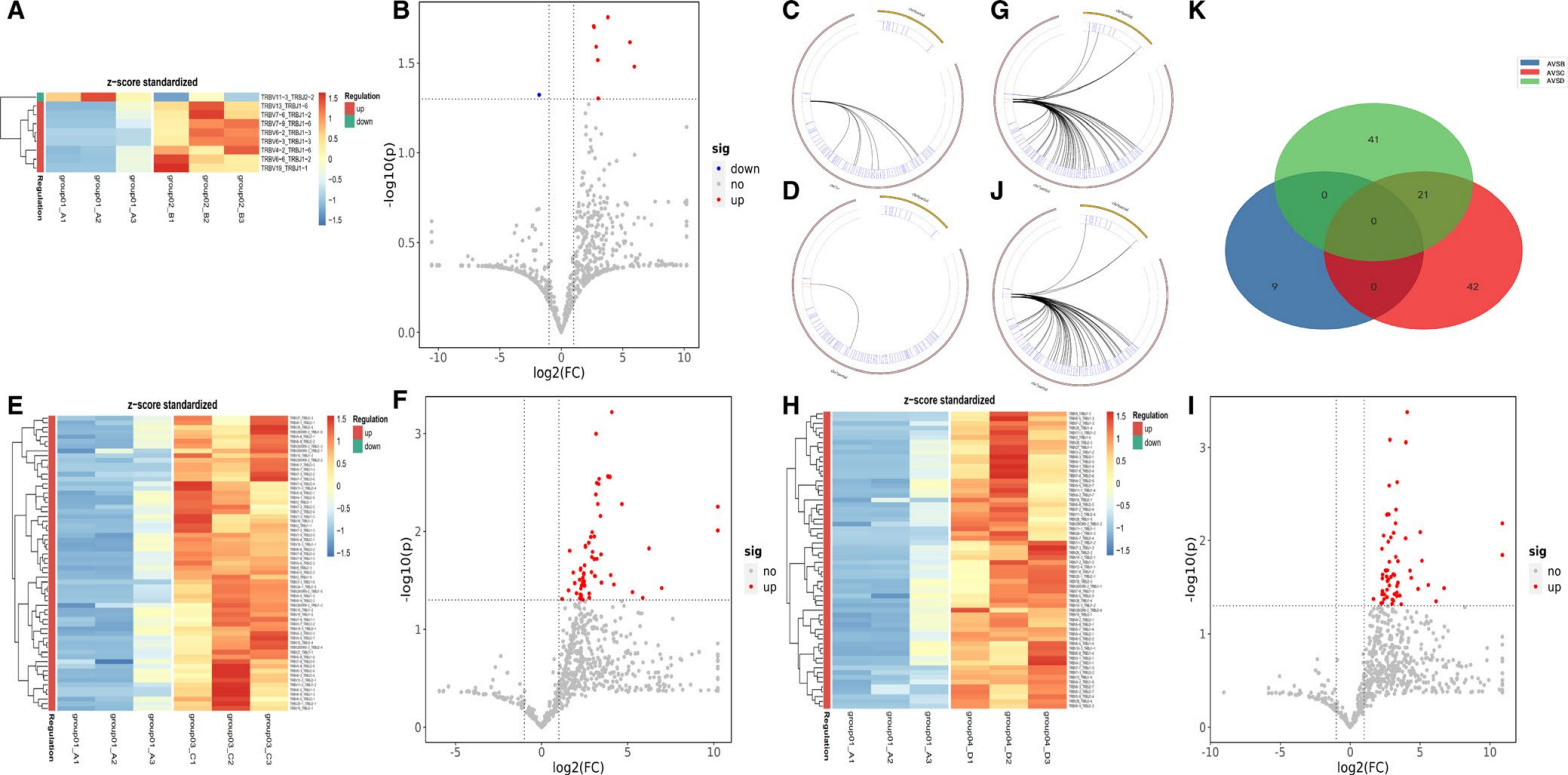
Different gene expression of V‐J gene combinations in four groups. (A, E, H) Heatmap of statistically significant differential V‐J gene combinations in group A VS B,group A VS C and group A VS D (|log2(FC) |> 1, *P* <.05). (B, F, I) The volcano plot showing differences in gene expression of V‐J gene combinations in group A VS B, group A VS C and group A VS D. (C) Loop diagram of up‐regulated V‐J gene combinations in group A VS B. Blue and red lines: TRBV, TRBJ; brown lines: recombination of TRBV and TRBJ. (D) Loop diagram of down‐regulated V‐J gene combinations in group A VS B. Blue and red lines: TRBV, TRBJ; brown lines: recombination of TRBV and TRBJ. (G, J) Loop diagram of up‐regulated V‐J gene combinations in group A VS C and group A VS D. Blue and red lines: TRBV, TRBJ; brown lines: recombination of TRBV and TRBJ. (K) Wayne diagram of meaningful differential VJ paired genes between 3 groups

We further analysed and compared the expression of VJ paired genes between the 4 groups. We found that compared with CP patients, there were significantly 8 down‐regulated genes (Figure 3A–C) and 1 up‐regulated gene (TRBV11‐3_TRBJ2‐2) in PC patients (Figure 3A,B,D). Meanwhile, compared with PC patients, the expression of 63 VJ paired genes was increased in PNET patients (Figure 3E,F,G) and 62 in PCL patients (Figure 3H,I,J). Upon analysis, we found that only 21 differential genes were overlapped in the comparison between PC patients and PNET or PCL (Figure 3K).

**FIGURE 4:**
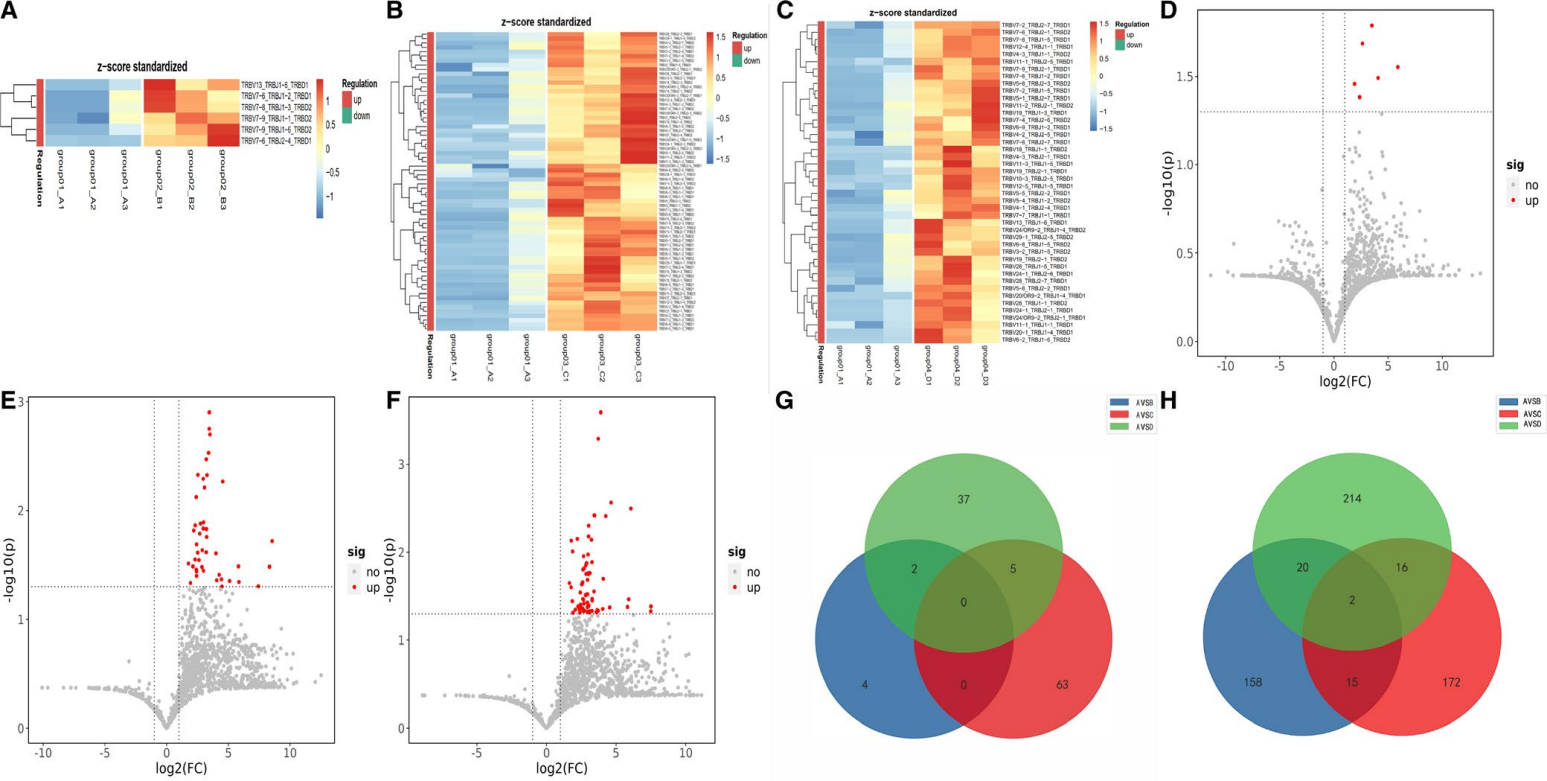
Different gene expression of VDJ gene combinations in four groups. (A, B, C) Heatmap of VDJ gene combinations in group A VS B, group A VS C and group A VS D (|log2(FC) |> 1, *P* <.05). (D, E, F) The volcano plot showing differences in gene expression of VDJ gene combinations in group A VS B, group A VS C and group A VS D. (G) Wayne diagram of meaningful differential VDJ gene combinations among groups: two overlapped genes (TRBV13_TRBJ1‐6_TRBD1, TRBV7‐6_TRBJ1‐2_TRBD1) in group A VS B and group A VS D; 5 gene combinations (TRBV5‐1_TRBJ27_TRBD1, TRBV11‐2_TRBJ2‐1_TRBD2, TRBV19_TRBJ2‐1_TRBD2, TRBV3‐2_TRBJ15_TRBD2 and TRBV28_TRBJ2‐7_TRBD1)were overlapped in group A VS C and group A VS D. (H) Wayne diagram of meaningful differential CD3 clonotypes among groups. Two common differential gene fragments: CSVIDQILWNEQF and CSARKRSSYNSPLHF

The D gene segment is located between V region and J region, intercepted from the end of the V zone to the beginning of the J zone. Analysing VDJ gene combinations further narrowed down the scope and compared the difference of TCR diversity between PC and other pancreatic diseases. We found that compared with PC patients, the expression of 6 VDJ gene combinations was significantly enhanced in CP patients (Figure 4A,D), 62 gene fragments in PNETs (Figure 4B,E) and 44 gene fragments in PCL (Figure 4C,F). However, there were no significantly up‐regulated gene fragments in PC patients compared with other pancreatic diseases. As demonstrated by Figures 2, 4 2 differential gene combinations were overlapped in the comparison between PC patients and CP or PCL (TRBV13_TRBJ1‐6_TRBD1, TRBV7‐6_TRBJ1‐2_TRBD1), and 5 differential gene combinations (TRBV5‐1_TRBJ27_TRBD1, TRBV11‐2_TRBJ2‐1_TRBD2, TRBV19_TRBJ2‐1_TRBD2, TRBV3‐2_TRBJ15_TRBD2 and TRBV28_TRBJ2‐7_TRBD1) were overlapped in the comparison between PC patients and PNET or PCL.

We also analysed the differences of Vβ, Jβ gene segments, V‐J and VDJ gene combinations among three groups except PC, as shown in Figures S2, –S4. There was no significant difference in the frequency of Vβ and Jβ genes between CP and PNET (Figure S2A,B,C,D), CP and PCL (Figure S3A,B,C,D), PNETs and PCLs (Figure 4A,B,C,D). We found that compared with CP patients, there were significantly 2 down‐regulated VJ paired genes and 27 up‐regulated genes in PNET patients (Figure S2E), and 28 up‐regulated and 1 down‐regulated gene in PCL (Figure S3E). Meanwhile, compared with PCL patients, the expression of 7 VJ paired genes was increased in PNET patients (Figure S4E). By further analysis of VDJ gene combinations, we found that compared with CP patients, the expression of 36 VDJ gene combinations was significantly reduced in PNET patients (Figure S2F), and 28 gene fragments were down‐regulated in PCL (Figure S3F). As for the comparison between PNET and PCL, we found only 2 up‐regulated genes and 8 down‐regulated gene fragments in PNET (Figure S4F).

These results suggested that TCRβ repertoires in PC patients were more oligoclonal than other pancreatic diseases. Nevertheless, there were fewer significant differential gene fragments among the other three types of pancreatic diseases.

### 3.4 Some significant differences in CDR3 amino acid clonotypes between PC and other pancreatic diseases

**FIGURE 5:**
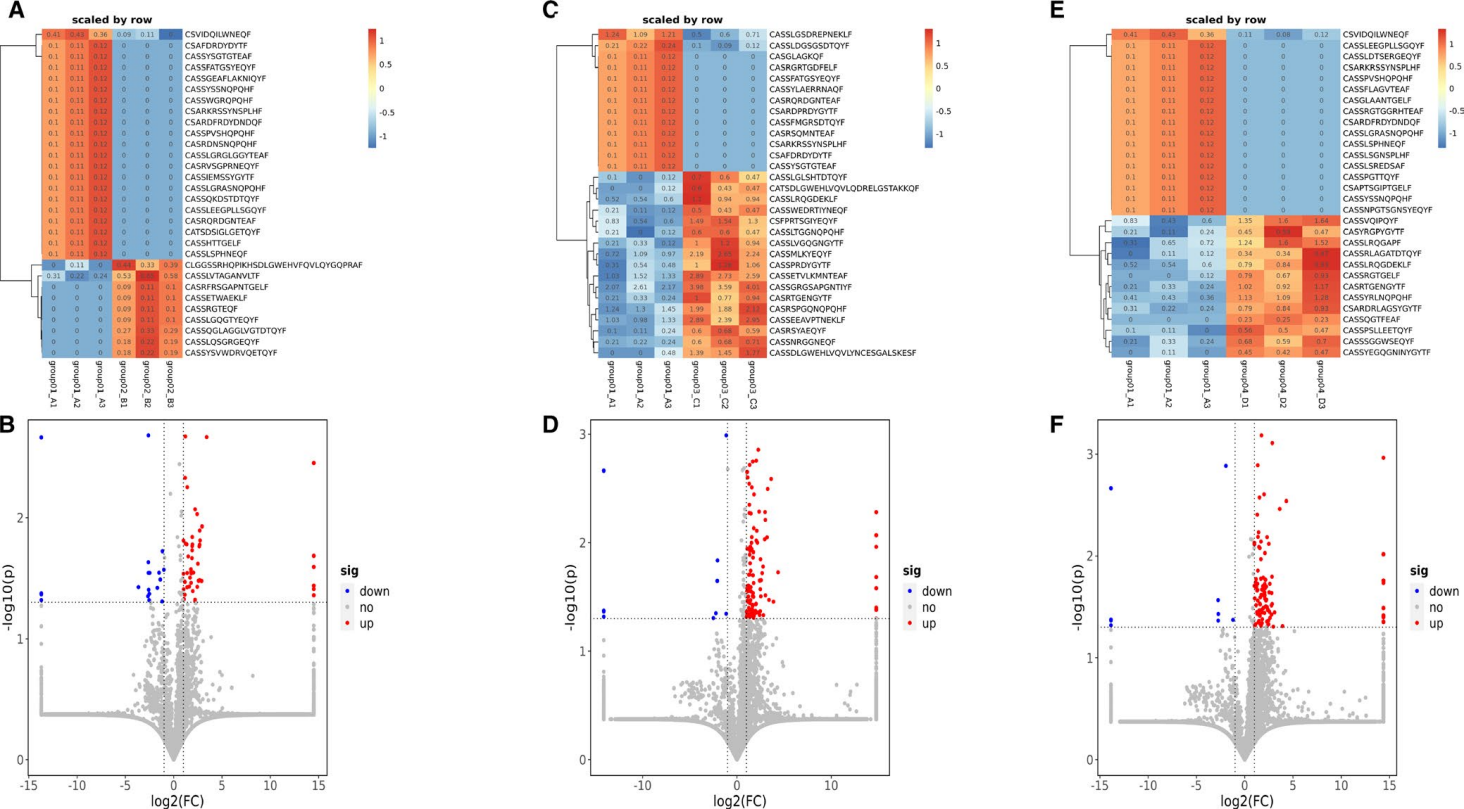
Different gene expression of CD3 clonotypes in four groups. (A, C, E) Heatmap of CD3 clonotypes in group A VS B, group A VS C and group A VS D(|log2(FC) |> 1, *P* <.05). (B, D, F) The volcano plot showing differences in gene expression of of CD3 clonotypes in group A VS B, group A VS C and group A VS D

After the V and J genes were determined, the CDR3 amino acid (AA) sequence was determined by the last amino acid ‘C’ (cysteine) of V gene and the first amino acid ‘F’ (phenylalanine) of J gene. The data showed that there were significant differences in CDR3 AA clonotypes between PC and other pancreatic diseases, including 147 decreased clones in PC VS PC (Figure 5A,B), 184 decreased clones in PC VS PNET (Figure 5C,D) and 226 down‐regulated clones in PC VS PCL (Figure 5E,F). Among the differential CDR3 AA clonotypes, 2 AA sequences were overlapped: CSVIDQILWNEQF and CSARKRSSYNSPLHF (Figure 4H). What's more, we found the number of some CDR3 AA clones was higher in PC patients compared with other pancreatic diseases. However, none of the up‐regulated AA clonotypes were overlapped.

Complementarily, we analysed the changes of CDR3 AA clonotypes in the remaining three groups except PC as shown in Figure S1–S3. We found 117 clones enhanced and 49 reduced in CP VS PNET (Figure S2G), 150 clones enhanced and 40 reduced in CP VS PCL(Figure S3G), 28 clones enhanced and 57 reduced in PNET VS PCL(Figure S4G).

Overall, there were significant differences regarding the diversity of TCR CDR3 immune repertoire between PC and other pancreatic diseases.

### 3.5 The diversity and similarity between PC and other pancreatic diseases using Simpson index

**FIGURE 6:**
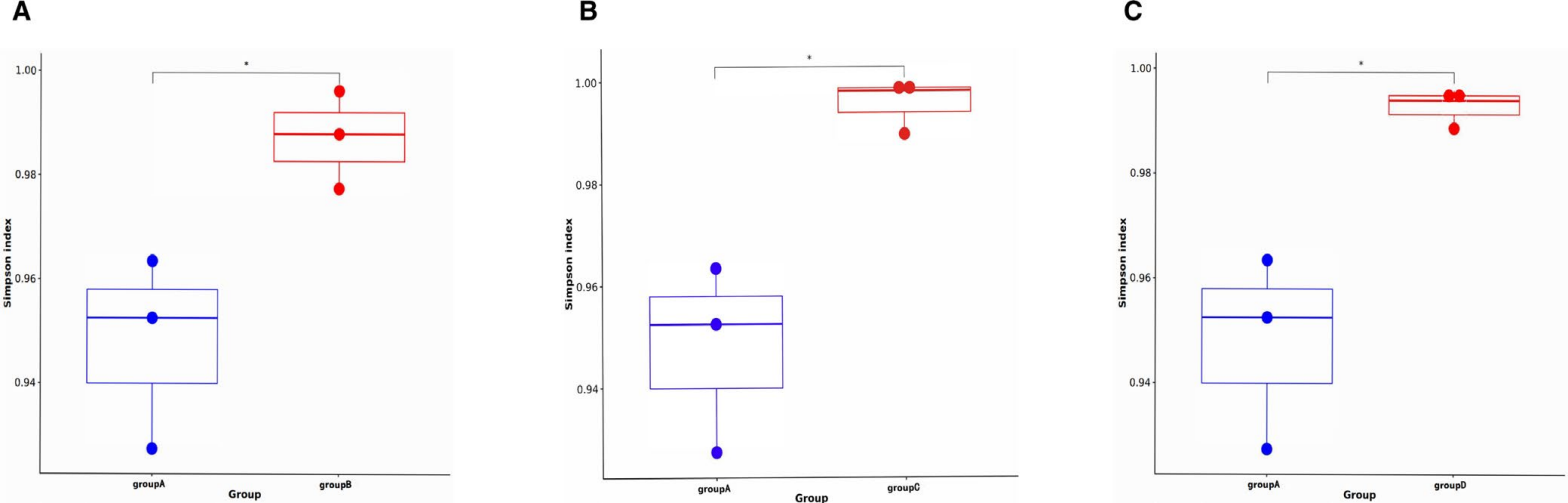
The diversity between 3 compared groups. Comparison of TCR CDR3 diversity by Simpson index (A, B, C) in group A VS B, group A VS C and group A VS D (*P* <.05)

Simpson index was used to analyse the difference of TCR CDR3 between PC and other pancreatic lesions. As shown in Figure 6, the Simpson index of TCR CDR3 in PC patients was significantly lower than that in other pancreatic diseases, demonstrating that the diversity was obviously different between PC and other pancreatic diseases. Nevertheless, there was no significantly different diversity among other pancreatic diseases (Figures S2H, 3H, 4H).

## 4 DISCUSSION

PC is a kind of extremely dangerous and highly malignant digestive tract tumour, whose prognosis is very poor.^29^ Due to the insignificantly early specific symptoms of PC and the lack of effective screening methods, most patients are diagnosed at an advanced stage, missing the best opportunity of treatment.^29^ Therefore, the cost‐effective methods for early diagnosis of PC are urgently needed. As in known to all, the immune system plays crucial roles in anti‐cancer activities during tumour development, of which T cells are the main components. And the characteristics and dynamic changes of the TCR repertoire during disease progression can be clearly profiled using deep‐sequencing. Currently, our understanding of the TCR repertoire of pancreatic diseases, especially PC, is extremely limited. TCR patterns are heterogeneous, and there are differences between regions within the same tumour in pancreatic cancer and many other malignancies.^2^, ^3^ Due to the low rate of radical resection for pancreatic cancer patients, especially those at advanced stages, it is difficult to obtain tumour tissues. Therefore peripheral blood samples are attractive alternatives to overcome these problems.

Our study was the first to use high‐throughput sequencing to evaluate the clonal composition of TCR in peripheral blood of different pancreatic diseases. We have not analysed the TCR repertoire of matched tumour tissues in our cohort of patients. However, Bai et al demonstrated that there were true differences between TCR repertoires of PC tissues and peripheral blood.^30^ In this research, we found that the TCR diversity was significantly decreased compared with pancreatic diseases, consistent with some other malignancies reported before.^31^ As a result, the TCR patterns of circulating T cells could reflect the immune status of pancreatic cancer and other pancreatic diseases in some aspects, although various from tumour‐infiltrating T cells.

Here, in our study we explored the TCR repertoire of peripheral blood cells from patients with different pancreatic diseases, including PC. The 4 groups revealed varied TCR repertoires in terms of CDR3 clonotypes, diversity, Vβ, Jβ gene segments and V‐J, V‐D‐J gene combinations. Through the analysis of deep‐sequencing data, we found the TCR diversity of PC patients was lower than other pancreatic diseases, indicating that the TCR composition of PC was oligoclonal. Besides, the fact that all PC patients included in this study were at early stage (TNM stage I/II) elucidated that the CDR3 diversity changed significantly in an early phase of pancreatic carcinogenesis, providing a new direction for early detection of PC.

Immunotherapy is a promising method for treating advanced PC, and remarkable progress has been made in adoptive T‐cell therapy and so an.^32^, ^33^ T cells can stimulate tumour‐infiltrating lymphocytes (TIL) to activate the adaptive immune system and infiltrate a large number of solid tumours, thereby killing tumour cells.^34^ The limited efficacy of immunotherapy in PC may be related to the unique immunosuppressive microenvironment with low mutation rate and low tumour T‐cell infiltration. Here in our study, the low‐level diversity of TCR repertoire might explain why most PC patients presented poor response to immunotherapy. It has been pointed out that the overall survival rate (OS) of PC patients with high number of tumour‐infiltrating lymphocyte (TIL) in CD8 + and PD‐1 + is significantly higher than that in the control one.^34^ Researchers also reported that clonality changes and clonal expansion in T‐cell library can predict the prognostic response of anti‐CTLA‐4 and other immunotherapy in PC patients.^35^ In our analysis of CDR3 diversity, we found that some of the AA clonotypes were significantly up‐regulated in PC patients, indicating that peripheral blood T cells in PC patients may have specific immune responses to some specific antigen targets, although further evaluation is needed to test the hypothesis. Studies have shown that PC is not totally an immunologically ‘cold’ tumour.^23^ Therefore, if we could develop a method to target this specific antigen or isolate this antigen‐specific TIL, the corresponding adoptive cell therapy may be effective for treating these patients.

There are still some aspects that need to be improved in this study. In our study, we only analyse β chain to represent the characteristics of whole TCR repertoire. Previous studies have shown that TCR composed of α chain and γδ chain besides β chain. A broader analysis of these chains may provide more comprehensive conclusions. In addition, if we can use flow cytometry technology to separate CD4 + and CD8 + T cells in PMBC and then sequenced respectively, the results are more reliable and accurate. Finally, we need to expand the sample size to verify our conclusion.

In summary, to the best of our knowledge, this is the first study to use high‐throughput sequencing to evaluate the clonal composition of TCR in peripheral blood of different pancreatic diseases. We found that there were significant differences related to TCR repertoire between PC and other pancreatic diseases, and PC is a relatively immunosuppressive tumour. Changes of peripheral TCR repertoire may be used to predict the progression of PC and the response to immunotherapy. And there may exist novel‐specific antigens in PC patients which could be used to design targeting immunotherapy in the nearly future.

## CONFLICT OF INTEREST

The authors declare that they have no conflicts of interest.

## AUTHOR CONTRIBUTION

Hui Wang: Writing‐original draft (equal). Yue Yuan: Data curation (equal). Chenglin Lu: Data curation (equal). Siqi Zhou: Writing‐original draft (supporting). Yixuan Zhang: Methodology (supporting). Jing Zhao: Software (supporting). Chenghu Xu: Data curation (supporting). Jie Yang: Data curation (supporting). Haochen Su: Writing‐review & editing (supporting). Borui Li: Writing‐review & editing (supporting). Xihan Li: Writing‐review & editing (supporting). Pin Wang: Software (supporting). Guifang Xu: Writing‐review & editing (supporting). Lei Wang: Writing‐review & editing (supporting). Xiaoping Zou: Methodology (supporting). Shanhua Bao: Project administration (equal). Shu Zhang: Data curation (equal); Project administration (equal). Ying Lv: Project administration (equal); Writing‐review & editing (equal).

## Supporting information

Fig S1

Fig S2

Fig S3

Fig S4

## Data Availability

The data used to support the findings of this study are available from the corresponding author upon reasonable request.
